# Genetic and Other Determinants for the Severity of Coccidioidomycosis: A Clinician’s Perspective

**DOI:** 10.3390/jof9050554

**Published:** 2023-05-11

**Authors:** John N. Galgiani, Amy P. Hsu, Daniel A. Powell, Jatin M. Vyas, Steven M. Holland

**Affiliations:** 1 Valley Fever Center for Excellence, College of Medicine-Tucson, University of Arizona, Tucson, AZ 85721, USA; danielpowell@arizona.edu; 2 Department of Medicine, College of Medicine-Tucson, University of Arizona, Tucson, AZ 85721, USA; 3 Department of Immunobiology, College of Medicine-Tucson, University of Arizona, Tucson, AZ 85721, USA; 4 BIO5 Institute, University of Arizona, Tucson, AZ 85721, USA; 5 Laboratory of Clinical Immunology and Microbiology, National Institute of Allergy and Infectious Diseases (NIAID), NIH, Bethesda, MD 20892, USA; twins@niaid.nih.gov (A.P.H.); sholland@niaid.nih.gov (S.M.H.); 6 Division of Infectious Diseases, Massachusetts General Hospital, Boston, MA 02114, USA; jvyas@mgh.harvard.edu; 7 Department of Medicine, Harvard Medical School, Boston, MA 02115, USA

**Keywords:** coccidioidomycosis, genetic variants, pathogenesis, immunity, fungal infections, human disease, mice

## Abstract

The endemic fungal infection, coccidioidomycosis, occurs after inhalation of one or very few *Coccidioides* spp. spores. Infections produce diverse clinical manifestations, ranging from insignificant to extremely destructive, even fatal. Approaches to understanding this range of consequences have traditionally categorized patients into a small number of groups (asymptomatic, uncomplicated self-limited, fibro-cavitary, and extra-thoracic disseminated) and then looked for immunologic differences among them. Recently, variants within genes of innate pathways have been found to account, in part, for infections that result in disseminated disease. This discovery raises the very attractive theory that, in patients without severe immunosuppression, much of the disease spectrum can be accounted for by various combinations of such deleterious variants in innate pathways. In this review, we summarize what is known about genetic determinants that are responsible for the severity of coccidioidal infections and how complex innate genetic differences among different people might account for the spectrum of disease observed clinically.

## 1. Introduction

Coccidioidomycosis (CM), often referred to as Valley fever because it was found to be endemic in the Southern San Joaquin Valley of California [1], is an increasingly important systemic fungal infection in the United States [2,3], elsewhere in the Western Hemisphere [4], and, because of travel, virtually anywhere else in the world [5]. The fungi *Coccidioides immitis* or *Coccidioides posadasii*, which cause CM, reside sparsely in the soil of endemic regions [6]. When the soil is disrupted, single-cell fragments of mycelia (arthronidia) are dispersed and may remain airborne for long periods of time and potentially travel over extensive distances [7]. Reported infections to the Centers for Disease Control and Prevention have ranged from as high as 22,000 cases in 2011. However, epidemiological models suggest that this underestimates the actual numbers of coccidioidal illnesses by 6- to 14-fold [8], and recent data support these calculations [9]. Although immunosuppression and pregnancy greatly heighten the chances of extra-thoracic disseminated coccidioidomycosis (DCM), severe and progressive spread of infection beyond the lungs [10,11,12] remains very uncommon. Most patients who develop DCM are actually not within recognized immunocompromised groups, leaving what exactly accounts for this lack of control an unexplained characteristic of a major proportion of cases of Valley fever.

In this review, we will describe what is known about factors that might explain the different disease patterns in patients. This review was stimulated by the recently published observations relating to polymorphisms identified in patients with DCM [13]. It is hoped that the clinical perspective followed in this description will stimulate additional studies to better understand these risks.

## 2. The Spectrum of Coccidioidomycosis in Humans

**Figure 1:**
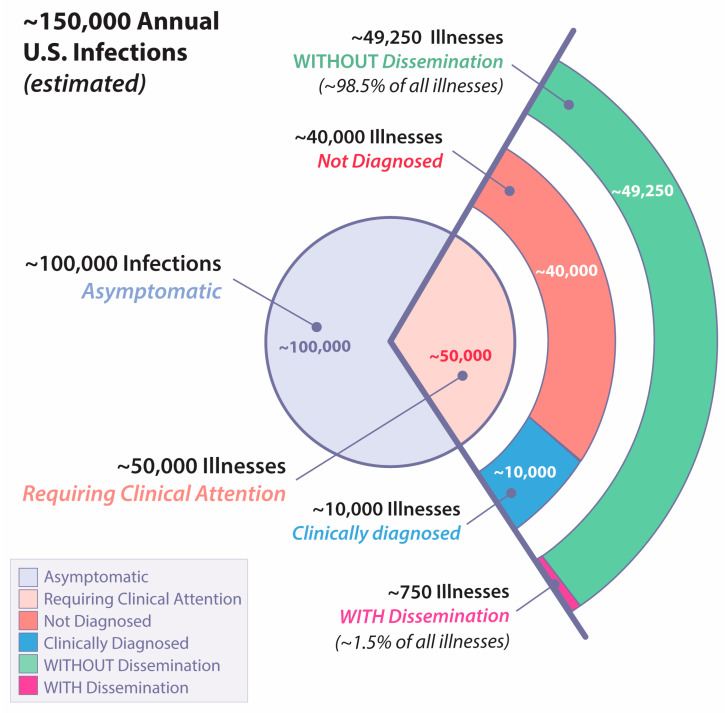
Estimated annual United States cases of coccidioidomycosis with different forms of illness. Percentages with and without dissemination extrapolated from [20].

In virtually all cases, CM occurs as the result of inhaling an airborne arthroconidium. While it is possible to be exposed to a relatively large inoculum [14], indirect evidence suggests most infections are likely initiated by one or a very small number of spores. For example, in mice, five or fewer arthroconidia can produce lethal CM [15]. Furthermore, when radiographic evidence for pneumonia is found in humans, it usually involves only a single lobe [16,17,18]. Finally, patients who develop CM appear to reflect the entire population within an endemic region, and occupations that afford high exposure to potentially infected dust account for a relatively small proportion of all CM that comes to medical attention [19]. Given the relatively uniform low inoculum size from naturally acquired CM, it is striking that the consequent spectrum of resulting disease is very wide ranging, traditionally categorized as four different types: asymptomatic infection, uncomplicated primary infection, fibro-cavitary pulmonary infection, and extra-thoracic dissemination. As shown in Figure 1, of the estimated 150,000 coccidioidal infections that occur annually, only about 0.5% of all infections result in dissemination.

### 2.1. Asymptomatic Infection

During World War II, prospective studies of large numbers of military troops training in the Central California valley convincingly demonstrated that approximately 60% of all persons who developed delayed hypersensitivity to a coccidioidal skin test preparation, had evidence of an intercurrent infection, and did not go on to have clinical illness [21]. In those studies, personnel were categorized as having asymptomatic infections only after intercurrent illness was excluded by careful interrogation.

### 2.2. Uncomplicated Primary Infection

Of those described in the World War II studies who were symptomatic, most developed an illness consisting of respiratory, musculoskeletal, dermatologic, and systemic complaints that lasted weeks to months but eventually resolved. This syndrome has been described in several more recent publications [22,23,24,25]. With the advent of fluconazole and other oral azole antifungal drugs, some of the patients with early infections and no apparent complications are now treated with these agents for possible benefit, even though the pivotal evaluation trials of efficacy have never been completed [26]. Furthermore, a few patients so treated have subsequently developed disseminated coccidioidal infections years after the early treatment was stopped [25,27]. Although this practice adds some uncertainty to the conclusion that these patients have a “self-limited illness,” it is likely that most would have controlled their infections without treatment based upon the prior epidemiology.

### 2.3. Fibro-Cavitary Pulmonary Complications

Pulmonary infiltrates in CM usually resolve to the point of being undetectable by routine radiographic imaging or computed tomography of the chest. A small percentage remains visible as stable nodules. These are often impossible to distinguish from cancer without histologic examination [28], but otherwise do not affect patient health even though they contain viable organisms [26]. More problematic are areas of pulmonary involvement, which result in tissue necrosis, liquefaction, and subsequent drainage into the airway, resulting in a pulmonary cavity. These lesions range from thin-walled and causing no symptoms to more complex anatomy involving areas of infiltration and fibrosis surrounding the cavity and variously associated with productive cough, chest pain, and hemoptysis. Prior to the advent of fluconazole and other azole antifungals [29], some of these lesions were progressive and debilitating. Currently, they are more effectively managed by long-term antifungal suppression.

### 2.4. Extrathoracic Dissemination

The exact likelihood that a coccidioidal infection will result in the hematogenous seeding of infection beyond the lungs, creating destructive lesions, is difficult to precisely determine. In 2007, there were 4832 persons with CM reported to the Arizona Department of Health Services, and a cohort was created of approximately every tenth person [30]. In a subsequent analysis, 26 of 324 (8.0%) of those persons were determined to have disseminated infection by medical record review [31]. If clinical illness accounts for a third of all coccidioidal infections and if clinical infections are under reported by as much as a factor of five [9], then the likelihood of any coccidioidal infection resulting in disseminated disease would be ~0.5% (Figure 1). The lesions within this group range from cutaneous, either single or multiple, to destruction of bones or joints, to chronic meningitis. If diagnosis or treatment is delayed, some of these lesions may wax and wane for years while others are relentlessly progressive [31], and coccidioidal meningitis is nearly always fatal without treatment [32,33].

## 3. What Is Known about Genetic Determinants of Susceptibility to Coccidioidomycosis

Despite the clear risk that cellular immunosuppression poses, most people who develop disseminated infections are not overtly immunocompromised, either by co-morbidities or therapies. In a 2017 review of all published case reports of DCM, of 318 cases that did not include those occurring during pregnancy, only 79 (25%) were immunosuppressed because of cancer, HIV, organ transplant recipients, or corticosteroids [34]. NIH-Mycoses Study Group antifungal clinical trials enrolled subjects with DCM, and the proportion of subjects that were considered immunocompromised was overall 17% (Table 1). CM diagnosed during pregnancy, especially in the third trimester, has been associated with high rates of dissemination [12], potentially due to pregnancy-associated immunosuppression. However, the overall rate of diagnosis of CM during pregnancy of any sort is very low [35], suggesting that the actual risk of severe disease is not as high as the reported cases might suggest.

Clinical observations suggest that the risks of DCM have a genetic basis. Most often, increased risk has been inferred from self-identified race/ethnicity (SIRE), especially among those who identify as African–American or Filipino. In military studies, 12% of coccidioidal infections in black soldiers were disseminated as compared to 1% in white soldiers [20]. In military personnel and their dependents (virtually all adults 21 years of age or older) for whom living conditions and duties were similar, dissemination occurred in 23.5% of clinically apparent CM in blacks, and 21% in Filipinos, compared with 2.3% among whites [39]. In a CM outbreak that resulted from an enormous California dust storm, rates of disseminated infections were 4.8-fold higher in blacks and 3.4-fold higher in Asians as compared to whites [7,40]. In the Arizona surveillance study referred to above [20], 16 of 257 (6.2%) white Arizonan coccidioidal infections were disseminated as compared to 5 of 20 (25%) black Arizonans, a four-fold difference. These estimates have been difficult to interpret because of their retrospective nature and the potential confounding effects of referral biases [41]. In addition, SIRE is not a precise genetic marker [42,43]. Studies of red blood cell ABO groups identified groups B and AB as being significantly associated with DCM [44,45]. Of note, B blood groups are more frequent in both people of African and Filipino ancestry [46]. Although there is no evidence that blood groups per se are critical determinants of disseminated infection, this association is further evidence of genetic associations with risk.

Another risk factor for disseminated infection in humans has been male gender. This is reflected in the demographics of the drug treatment trials (Table 1), the California windstorm study [47], the Arizona study [20], and renal transplant recipients [45]. There has also been concern that the apparent gender association might reflect different degrees of exposure rather than genetic factors. However, in a retrospective study of non-human primates and veterinary patients in addition to humans, there were also overall higher rates of infection and severity in males, suggesting that these observations are not determined by exposure alone [48].

**Figure 2:**
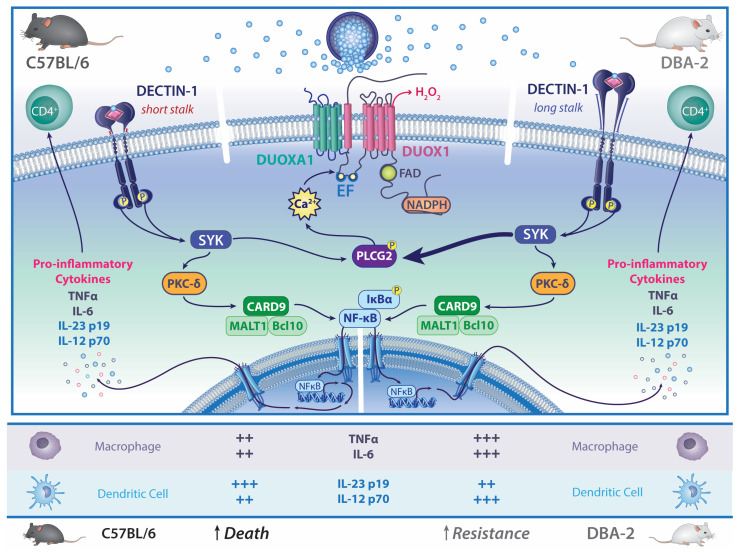
Differential responses to coccidioidal infection in mice. C57BL/6 mice are highly sensitive to coccidioidal infection and possess *Dectin-1,* which encodes a short stalk. DBA-2 mice are resistant to coccidioidal infection and possess *Dectin-1* which encodes a long stalk. Expression of pro-inflammatory cytokines from immune cells stimulated by *Coccidioides* spp. differs between the two inbred strains of mice. The cytokine differences listed have been observed experimentally and are not the exclusive source of the cells listed. Illustration by Nicole Wolf, MS, ©2023.

Murine studies have increased our understanding of DCM through the use of inbred strains that have different susceptibilities to experimental coccidioidal infection. A survey of different strains identified C57BL/6 mice as highly susceptible (LD_50_ log_10_ 2.77± 0.33) while DBA/2 is much more resistant (LD_50_ log_10_ 5.25 ± 0.36) to intraperitoneal infection with *Coccidioides immitis*, strain “RS” [49]. Subsequent studies using an intranasal infection found a similar relationship in lung fungal growth (7.8 log_10_ colony forming units (CFU) in C57BL/6 mice versus 2.4 log_10_ CFU in DBA/2 mice at day 12 following infection), although the DBA/2 mice produced a very extensive inflammatory pneumonia that resulted in similar lethality, indicating that pathogenesis is not completely dependent on fungal proliferation [50]. Using lung CFU enumeration after intraperitoneal challenge and IL-10 expression as quantitative traits in C57BL/6 and DBA/2 mice, two loci were identified that were associated with resistance [51]. One of the loci on chromosome 6 was close to the *Clec* cluster of genes, which includes *Clec7a*, which encodes Dectin-1, the principal β-1,3-glucan receptor that is expressed on a variety of cells, including epithelial and myeloid cells. Studies of C57BL/6 with *Clec7a*/DECTIN-1 deletion as well as the cross with DBA/2 mice demonstrated the importance of Dectin-1 in conferring resistance to intranasal coccidioidal infection [52]. When the expression of Dectin-1 was examined in C57BL/6 and DBA/2 mice, it was found that DBA/2 mice expressed full-length (long-stalk) Dectin-1, whereas C57BL/6 mice spliced out exon 3, which encodes most of the stalk, resulting in short-stalk Dectin-1 [53]. In vitro, when stimulated with formalin-killed spherules, DBA/2 macrophages produced more TNF and IL-6 than macrophages from C57BL/6 mice, and the amount of TNF made was dependent on Dectin-1. In addition, myeloid dendritic cells from C57BL/6 mice made more IL-10 and less IL-23p19 and IL-12p70 than DBA/2 dendritic cells. These responses were blocked by a monoclonal antibody to Dectin-1. Taken together, these results support a functional difference between long- and short-stalk Dectin-1 that accounts in part for the greater susceptibility of C57BL/6 mice compared to DBA/2 mice to coccidioidal infection (Figure 2). However, backcrossing the C57BL/6xDBA/2 F1 with C57BL/6 mice resulted in 34 progeny that were tested for susceptibility to intraperitoneal coccidioidal infection [51]. Using frozen lung samples from those progenies, each was characterized for its expression of either long- or short-stalk Dectin-1. When Dectin-1 isoform expression was compared to susceptibility to infection, there was only a 75% correlation, indicating that other genes in addition to Dectin-1 are likely involved in susceptibility to infection. 

A limitation of these experimental murine infections is that the coccidioidal strains used, the “RS” strain of *C. immitis* and the Silveira strain of *C. posadasii*, are rapidly lethal in both C57BL/6 mice due to fungal proliferation and in DBA/2 mice due to the excessive inflammatory response, neither of which is the typical course of human coccidioidal infections. Although an early survey demonstrated that the virulence of many different coccidioidal strains showed considerable variation [54], these two fungal strains were selected because the model was primarily designed to evaluate the efficacy of drugs and vaccines [55], and a rapidly lethal infection was preferred for those studies. Recently, the murine infection model has been improved by using a clinical isolate, strain 1038 of *C. posadasii* (Cp1038), that is lethal for C57BL/6 but only after six or more weeks [56]. Moreover, infection of C57BL/6xDBA/2 mice with Cp1038 produces an arrested infection with viable fungal organisms within well-formed pulmonary granulomata. Both of these outcomes are much more in keeping with the behavior of CM in humans, and it would be interesting to examine the genetics of murine susceptibility using this particular infection model.

An interesting convergence on Dectin-1 has recently been observed in an analysis of patients with different forms of coccidioidal infection [57]. In 67 patients with DCM evaluated by whole exome sequencing, variants for genes involved in Dectin-1 pathway signaling were overrepresented as compared to those in the Genome Aggregation Database (gnomAD). These included *CLEC7A* c.714T>G, p.Y238* (encoding DECTIN-1; both homozygous and heterozygous), and variants in *PLCG2* (encoding PLCγ2). Further downstream from Dectin-1, variants in DUOX1/DUOXA1 were also found. A second, independent cohort of patients with disseminated infections demonstrated these same associations. Only two of the 67 patients in the original discovery cohort possessed rare deleterious haploinsufficient mutations in *STAT3*, distinct from the previously recognized dominant negative or gain of function mutations in that gene. Excluding these, 49% of the disseminated infections had one or more variants related to Dectin-1 signaling.

Although the original experimental cohort was comprised only of patients with disseminated coccidioidal infection, the second validation cohort also had 59 patients with chronic pulmonary infections. This group of patients had radiographic evidence of infiltrates for more than one year. Repeating the ancestry matching, *CLEC7A* remained significantly overrepresented, with some but not all other variants at or near levels of significance. In a similar analysis, we also included 363 patients with uncomplicated primary pneumonia, derived from both Arizona and California cohorts. When examining these individuals to determine infection risk unassociated with dissemination, no gene that reached overrepresentation in the validation cohort was significant, indicating that the genetic control of infection per se differs from that of containment. In the aggregate, these studies suggest that variants in the *CLEC7A/PLCG2/DUOX1/DUOXA1* pathway are not risk factors for primary infection but rather for control of infection (*CLEC7A*) and dissemination (*PLCG2/DUOX1/DUOXA1*).

## 4. Applying Current Knowledge Forward to a Unifying Model

**Figure 3:**
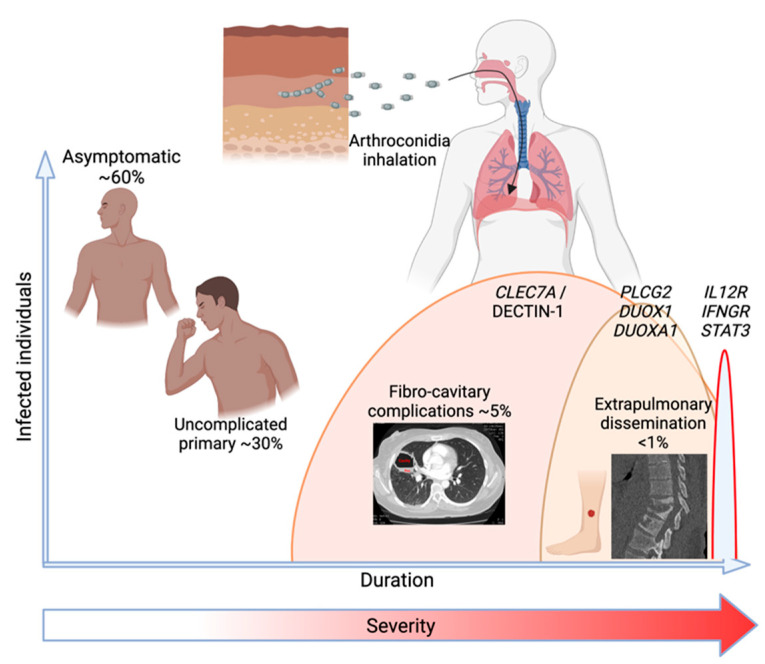
The range of clinical manifestations of human coccidioidomycosis and their overall frequency of occurance in relation to common durations of illness and severity and the several genetic variants associated with each. Created in BioRender.

As described earlier in this review, it has been traditional to categorize patients into four possible consequences of coccidioidal infection: asymptomatic, self-limited pneumonia, chronic fibro-cavitary pneumonia, and dissemination. Trying to understand pathogenesis started with this categorical description, and implicitly, each type of outcome would also have a mechanistic explanation particular to each group. Since profoundly depressed cellular immunity is a strong risk factor for disseminated infection in humans, some subtle impairment of acquired cellular immunity might be the explanation in patients without an obvious immunosuppressing condition. Indeed, only 12 patients with specific deleterious mutations in the IL-12/IFNg acquired cellular effector pathway, which is known to control intramacrophagic infection, have been described [34]. These mutations disrupt cellular signaling between IL-12/23 producing myeloid cells and IL12R expressing T and NK cells, leading to the production of IFNg. However, as is now clear [34,57], monogenic mutations in cellular mutations only explain a minute fraction of disseminated infections. Moreover, there have been no posited explanations for why some patients have no symptoms, others have self-limited infections, and still others develop progressive infections. Finding that DCM patients as a group have overrepresented deleterious polymorphisms of genes involved in the initial responses to coccidioidal infection invites a new perspective on how immunogenetic differences may explain the spectrum of coccidioidal disease (Figure 3). Furthermore, complex genetic interplay between polymorphisms in innate and acquired pathways could provide models more consistent with the several clinical characteristics that CM displays, as listed below.

For example, patients who never experience illness following exposure to a coccidioidal spore may have innate responses that competently reduce fungal proliferation such that the resulting inflammation is insufficient to generate respiratory symptoms, presumably leading to clearance or containment of the organism in granulomas. The cells involved may be alveolar macrophages within the terminal bronchioles or neutrophils and eosinophils recruited from the blood when spherules rupture. Since many of the systemic symptoms associated with early infection, such as arthralgias, myalgias, skin rashes, and profound fatigue, are systemic immunologic responses unrelated to the severity of the pulmonary infection [58], patients who are asymptomatic would also have such responses controlled.

In contrast, patients who develop symptoms but eventually resolve their illness without complications may have slightly less effective or rapid early innate responses. This impairment does not prevent the acquisition of effective cellular memory but is still insufficient to adequately control fungal proliferation prior to the activation of the memory response. Some symptomatic patients may have fungal proliferation greatly limited, while systemic signs and symptoms are apparently triggered by the antigenic stimulus [58]. For example, of the urgent care patients diagnosed with *Erythema nodosum* due to CM, almost none were also diagnosed with pneumonia [9].

In those patients who develop disseminated infection, defects in innate fungal recognition by myeloid cells or pulmonary epithelial cells result in impaired TNF responses to the rapidly multiplying fungus, resulting in slower local activation of sterilizing inflammation. This is the implication suggested by the recent immunogenetic studies [57]. In most patients, systemic immunity eventually does develop, but the delay permits the fungus to establish extra-thoracic sites that have a disrupted blood supply and other damage that prevents systemic immunity from effectively clearing it. This interplay of the delayed innate response in producing effective systemic immunity and the tissue damage that interferes with systemic immunity once it is established is especially attractive because it accounts for several of the clinical characteristics below.

The pathogenesis of fibro-cavitary pulmonary disease is the least well understood. There have not been genetic associations identified with these manifestations in the past. Rather, fibro-cavitary pulmonary complications are more common after puberty [59] and in patients with uncontrolled diabetes [60]. It is possible that the pulmonary complications have an acquired or metabolic explanation rather than an immunogenetic one [61].

## 5. Clinical Characteristics of Human Coccidioidomycosis Linked to Innate Signaling Defects

In addition to the broad spectrum of outcomes described in the previous section, there are additional features of this disease’s behavior that a model of coccidioidal pathogenesis must accommodate. In many ways, inadequacies or failures in the early responses to infection due to the interactions of multiple, relatively common genetic polymorphisms provide plausible explanations for these clinical observations.

### 5.1. Disseminated Disease very Rarely Occurs in Multiple Generations

To date, only a single family has been identified in which disseminated infection has occurred in more than one generation. In that report [62], disseminated infection was diagnosed in a grandmother, a mother, and a son. All three had the same mutation in *STAT4*, and a genetically engineered mouse with the same mutation demonstrated increased susceptibility to experimental infection. That multi-generational DCM is so infrequently recognized reinforces a model that does not rely on single gene mutations.

### 5.2. Clinically Significant Disseminated Disease Occurs as a Progression of the Initial Infection

It has been known since the epidemiology of CM was first described [20,63] that patients who develop destructive extrapulmonary lesions do so within the first weeks to months after the initial infection. This has been confirmed in a recent assessment of a pre-treatment cohort from a VA-Armed Forces natural history study [31]. Of 53 patients in whom the onset of infection was known, only five (9%) had their disseminated infection develop after six months. Defects in early responses to initial infection are very consistent with this pattern.

### 5.3. Patients Who Develop Disseminated Coccidioidomycosis Usually Have an Absent or Self-Resolving Pulmonary Illness

In one retrospective study of 150 DCM patients [64], 70 had no antecedent pulmonary illness, and for most of the rest, CM was first diagnosed by a biopsy of their extrapulmonary infection. This lack of clinically apparent pulmonary disease in DCM patients was also documented in another study [31].

### 5.4. Patients with Disseminated Infection Rarely if Ever Develop Pulmonary Illness from a Subsequent Respiratory Exposure to Coccidioides

That this is true for patients with uncomplicated and self-limited CM is a widely held belief, but it is nearly impossible to prove. There is one case of a person with a prior documented infection who had massive laboratory exposure [65] and subsequent serologic evidence of infection. However, even in that case, there was no residual disease. Similarly, there are no reports of patients with DCM subsequently developing lobar CM pneumonia, which would suggest a new respiratory coccidioidal infection.

### 5.5. Disseminated Disease can Reactivate as a Result of Profound Cellular Immunodeficiency

In patients with past infections, there are frequently, and perhaps nearly always, residual viable organisms present in the pulmonary or other sites. For example, residual pulmonary coccidioidal nodules, when biopsied or removed because of the concern for cancer, routinely yield fungal growth if cultured [66,67]. While in most people, these infections do not reactivate, protracted cellular immunosuppression results in a major reversal of that control. In patients with Hodgkin’s disease cared for at Stanford Medical Center, which is outside of the coccidioidal endemic region, the frequency of reactivation of CM increased with increasing intensity of chemotherapy [68]. Additionally, prior to effective anti-retroviral therapy for HIV, 279 (46.4%) of 602 patients with CM as their AIDS-defining opportunistic infection resided in areas where CM was not endemic [69]. In one case report, an AIDS patient in Spain developed CM twelve years after leaving the United States [70]. More recently, patients with severe SARS-CoV-2 infections treated for protracted periods with high doses of corticosteroids have occasionally developed active coccidioidal infections while still hospitalized, presumably contracted sometime prior [11]. Using the recently developed modification of the experimental murine model of CM referred to above, the stabilized infection created in C57BL/6xDBA/2 mice is abrogated by treatment with dexamethasone [56].

In persons who are normally immunocompetent, the prevention of disseminated disease by the successful early handling of infection is completely consistent with the critical role that acquired cellular immunity plays in the maintenance of that control.

### 5.6. While Biologic Response Modifiers Increase the Risk of Disseminated Coccidioidal Infection, Many Patients Treated with Biologic Response Modifiers Control Their Infection without Complications

Since the introduction of anti-TNF therapies for rheumatoid arthritis and other rheumatologic diseases, their potential for increasing the risk of serious coccidioidal infections has been an ongoing concern and has led to black-box warnings [10,71,72]. Recently, a retrospective study of patients with autoimmune diseases demonstrated that biologic response modifiers increased the risk of DCM by 2.4-fold [73]. Still, only 34.6% of patients receiving biologic response modifiers developed DCM compared to patients not receiving these therapies. Since this study reviewed charts of patients within Arizona, where coccidioidomycosis is endemic, it is not certain whether these infections were acquired before or after the biologic response modifiers were begun.

For biologic response modifiers that interfere with the function of discrete innate signaling molecules, the model proposed here helps understand the risks. For example, if a patient had existing cellular immunity to *Coccidioides* at the time anti-TNFα treatment was started, the risk of reactivation would be very low because the acquired CD4-mediated immunity would remain intact. In contrast, for patients with existing cellular immunity to *Coccidioides* who had untreated advanced HIV, the risk of disseminated infection would be high because of the progressive erosion of CD4 memory responses. As is the case with tuberculosis infection, anti-TNFα therapy would have a more profound effect on incident rather than prevalent disease [74].

Recent work has examined the effect of anti-TNFα treatment in the stabilized infection model described above [75]. When very high doses of anti-TNFα therapy were started at the time of the infection, C57BL/6xDBA/2 mice failed to gain control of the infection and succumbed to lethal disease, whereas isotype-treated controls survived with controlled disease. Interestingly, even if anti-TNFα therapy was used only for the first two weeks of the infection, mice were still unable to control the infection and succumbed in a time frame similar to those receiving continuous anti-TNFα therapy. Therefore, responses very early in infection are critical to the establishment of host control of *Coccidioides*. However, mice starting high-dose anti-TNF therapy after they had established control were still susceptible to infection. One way to reconcile these results with clinical observations is that anti-TNF treatment in humans is likely at significantly lower functional doses than were used in the murine studies. There appears to be a dose-related effect of anti-TNF therapy on the incidence of infectious complications in general [76,77,78].

### 5.7. Disseminated Infections That Recur following Discontinuation of Antifungal Therapy Usually Do so at a Previously Identified Site of Infection

In studies of oral azole antifungals to treat CM, relapses were common when treatment was stopped [79]. Most frequently, recurrence developed where the infection had previously been identified. This characteristic is used clinically to assess the risk of discontinuing treatment. For example, if the original site of dissemination was on the skin, where recurrence could be easily managed by re-instituting treatment, cessation of azole therapy is a manageable risk. On the other hand, if dissemination involved the central nervous system, the risk of relapse was very serious [80], leading to the recommendation of life-long treatment for coccidioidal meningitis [26]. These observations speak to the likelihood that systemic-acquired cellular immunity has developed in most patients, even though they developed extrapulmonary dissemination, and to the inability of that systemic immunity to eradicate disseminated lesions once established.

### 5.8. Non-Destructive Disseminated Infections Which Are Discovered Incidentally and Require no Medical Treatment also Occur

Coccidioidal infections of the genitourinary tract have been identified without associated signs or symptoms and are often managed without antifungal therapy [81,82,83]. Moreover, chorioretinal lesions have been reported to be damaging, and cultures have yielded fungal growth [84]. However, in a prospective study of 54 patients with prior primary untreated coccidioidal pneumonia, full eye examinations revealed that five (9%) had chorioretinal lesions without ocular destruction or interference with vision [85]. The pathogenesis model proposed here would account for these observations by assuming that those anatomic sites were sufficiently intact to allow acquired cellular immunity to be effective in arresting fungal proliferation, perhaps in the same way that residual pulmonary nodules, which frequently contain viable organisms, are stabilized without progression.

### 5.9. Patients with Disseminated Infection Occasionally Develop Dermal Hypersensitivity to Coccidioidal Antigens

Patients with disseminated infection frequently do not display dermal hypersensitivity to coccidioidal antigens, especially before active infection is controlled with antifungal therapies [86]. This also suggests that acquired cellular immunity may eventually be established in patients who previously disseminated their infections.

## 6. Other Factors That Might Modify the Genetics Model

While complex immunogenetic explanations might fully account for the broad spectrum of coccidioidal disease, there are other factors that have been largely unexplored.

The differences between the infectious organisms themselves have not been fully explored. In settings of relatively high inoculum exposure, such as outbreaks associated with construction or archeology excavations, clinical pulmonary illness in relatively short periods of exposure can occur at high frequency, although the risk of disseminated infection does not seem to increase [87]. However, what has not been determined is whether clinical illness of any sort is related to differences in fungal strains. In older studies, the virulence of isolates in mice was not well correlated with the illness experienced by the patient from whom the isolate was obtained [54]. However, very little has been conducted in this area since fungal genomics became available.

Another group of possible influences on the severity of coccidioidal illness are co-infections and comorbidities. We have mentioned the association between profound immunosuppression and diabetes as important risk factors [10,60]. Recently, there has been interest in the possible potentiation of CM by concurrent SARS-CoV-2 infection, but thus far little effect has been identified other than that associated with long-term high-dose corticosteroid treatment for COVID-19 and its deleterious consequences for CM [11]. The roles of Herpes viruses (CMV or EBV), Parvovirus 19 [88], and perhaps other co-infections that transiently interfere with normal immunologic responses have also not been evaluated. Similarly, there has been little examination of comorbidities other than diabetes or immunosuppressives. One retrospective review of adult cystic fibrosis pulmonary disease patients suggested that this group had a lower prevalence of CM than the general population [89]. There have been no similar studies of patients with asthma, chronic obstructive pulmonary disease, cigarette smoking, or other comorbidities that might alter the risk of either pulmonary or extra-thoracic complications.

There are likely other factors that influence the risk of or resistance to coccidioidal disease that have not been assessed. For example, the gut (and likely the airway) microbiota, can influence both autoimmunity and resistance to some infectious diseases [90,91]. Additionally, toxins and pollutants within the environment, which have numerous effects on health [92], might result in altered coccidioidal disease. Similarly, emotional stress and other cognitive conditions have demonstrated links to immunologic function [93]. None of these factors have been investigated in relation to the severity of CM.

## 7. Conclusions

In this review, we have proposed a framework for understanding the pathogenesis of CM that is consistent with both the clinical characteristics of CM in different patients and the newest information about genetic polymorphisms in innate immune pathways associated with DCM. Much more needs to be conducted to validate, refine, or refute this framework. As we learn more about the immunogenetic control of CM, it may also serve as a model for other complex genetic diseases.

## Figures and Tables

**Table 1:** NIH-Mycoses Study Group studies of antifungal drugs to treat disseminated coccidioidomycosis.

Study	Subjects		
	All	Immunocompromised	Male
Fluconazole for meningitis [36]	50	12 (24%)	41 (82%)
Itraconazole for non-meningeal dissemination [37]	26	1 (3.8%)	21 (81%)
Posaconazole for non-meningeal dissemination [38]	12	2 (18%)	8 (67%)

## Data Availability

Not applicable.
